# Trends in Opioid Prescribing for Nonspecific Low Back Pain After CDC Guidelines and California Prescription Drug Monitoring Programs, 2012-2024

**DOI:** 10.1177/00333549261486260

**Published:** 2026-10-25

**Authors:** Michelle W. Tong, Gabby B. Joseph, Sharmila Majumdar

**Affiliations:** 1 Department of Bioengineering, University of California, Berkeley, Berkeley, CA, USA; 2 Department of Bioengineering, University of California, San Francisco, San Francisco, CA, USA; 3 Department of Radiology and Biomedical Imaging, University of California, San Francisco, San Francisco, CA, USA

**Keywords:** nonspecific low back pain, opioid prescribing guidelines, pain management, prescription drug monitoring programs, interrupted time series

## Abstract

**Objective:**

Nonspecific low back pain (ns-LBP) is a widespread health challenge, and opioids were frequently prescribed upon first LBP presentation in the United States during the opioid epidemic. We assessed temporal changes in quarterly (Q) opioid prescriptions for new-onset ns-LBP from 2012 through 2024 at a California medical center amid evolving voluntary federal recommendations and mandatory state regulations.

**Methods:**

We analyzed opioid prescription rates (prescription within 6 months of diagnosis) for ns-LBP patients diagnosed in all departments and stratified by emergency department (ED), and we compared high- versus low-dose prescription (≥50 morphine mg equivalents/day). Change point detection identified transitions without policy priors, and interrupted time series regression quantified level changes and trend modifications.

**Results:**

Of 31 940 ns-LBP patients, 2077 (6.5%) received opioids (mean [SD] age, 51.0 [17.6] y; 45.3% male; 53.7% White). Change points occurred at 2015 Q2 and 2018 Q4. Prescribing odds declined 2.7% per quarter from 2012 Q1 through 2024 Q4 (95% CI, 0.4%-5.0%; *P* = .02). ED patients (16.5% prescribed opioids vs 5.8% non-ED) showed a 54.2% immediate reduction at 2015 Q2 (95% CI, 18.8%-74.2%; *P* = .008), declining from 27.4% to 12.2% before plateauing; non-ED patients showed a 42.8% reduction at 2018 Q4 (95% CI, 27.5%-54.9%; *P* < .001). High-dose prescribing remained unchanged among opioid recipients.

**Conclusion:**

Change points coincided with anticipatory adoption of the 2016 Centers for Disease Control and Prevention recommendations and 2018 California mandatory drug monitoring implementation. ED settings showed disproportionate reductions at 2015 Q2, consistent with knowledge diffusion of voluntary guidelines in settings with frequent opioid prescribing, while non-ED reductions coincided with immediate regulatory compliance at 2018 Q4. This distinction can guide future policy selection to address environment-specific adoption barriers.

## Introduction

Nonspecific low back pain (ns-LBP) is a widespread global health challenge, affecting approximately 557 million people of all ages, with a projected 36.4% increase in the next 30 years.^1,2^ For ns-LBP patients, pain management presents a significant and complex health care challenge due to limited high-efficacy treatments and diagnostic uncertainty.^3^ During the height of the opioid epidemic,^4^ opioids were frequently prescribed upon first LBP presentation in both emergency departments (EDs) (up to 60% from 1999 to 2015)^5,6^ and primary care (61% in 2004,^7^ up to 30% from 1999 to 2015^6^) in the United States. However, meta-analyses found that opioids for LBP management had limited or inconclusive benefits with statistically significant risks, including substance use disorders, overdose, and adverse mental health outcomes.^3,8,9^

US policy responses included updating guidelines and requirements to align opioid prescribing practices with emerging evidence on the increased risk of adverse effects. Major federal policy inflection points include the Centers for Disease Control and Prevention (CDC) guideline updates on opioid prescribing for chronic pain to limit high-risk opioid prescribing in 2016^10^ and to encourage individualized risk–benefit assessment in 2022.^11^ The 2016 CDC guideline identified several high-risk prescribing patterns associated with increased risk of overdose and opioid use disorder, including high daily opioid dose (≥50 morphine mg equivalents [MME]/day), concurrent opioid and benzodiazepine prescribing, multiple opioid prescribers, and long-duration opioid therapy.^10^ California state policy implemented Assembly Bill No. 2760 to offer naloxone to patients at elevated risk of opioid overdose and the Controlled Substance Utilization Review and Evaluation System (CURES) Mandatory Consultation to strengthen review of patients’ controlled substance histories before prescribing.^12-14^ These paradigm shifts coincided with measurable changes in prescribing patterns for LBP patients, including a decline in initial opioid prescription rate from 46%^15^ to 21.6% observed between 2009-2011 and 2017,^16^ prescription duration from 6.95 to 6.38 days in the second postguideline year,^17^ and prescription dose (≥50 MME/day) from a projected 22.5% to 21.1% by the second postguideline year.^17^

However, guideline adoption often occurs gradually due to systemic barriers, including the time required for health care provider education, institutional policy updates, and workflow modifications. Implementation efforts aim to bridge awareness gaps, overcome resistance to care changes, and accommodate increased time demands for comprehensive assessment at the patient, clinician, and institution levels.^12,18^ These challenges may introduce substantial lags between guideline release and measurable LBP practice changes, especially when alternative high-efficacy treatments are limited. Current evidence lacks robust temporal analysis that distinguishes policy effects from underlying secular trends in opioid prescribing patterns for ns-LBP. This analytical gap limits our understanding of guideline effectiveness and complicates efforts to design evidence-based interventions for future policy implementation.

To address this gap, we performed an interrupted time series analysis^19,20^ to evaluate the temporal associations between guideline releases and ns-LBP opioid prescribing patterns from 2012 through 2024. This established public health approach captures preexisting trends while isolating policy intervention effects, providing essential evidence for health care monitoring and informing future guideline implementation strategies.

## Methods

### Cohort Definition

We conducted a retrospective study using the University of California, San Francisco (UCSF) Information Commons, a deidentified electronic health records database. The UCSF Institutional Review Board determined that this study was exempt from formal IRB review because we used only deidentified data.

We identified patients with new-onset ns-LBP using keyword-based queries informed by clinical selection criteria (Figure 1; Supplemental Code 1-3). Inclusion criteria identified patients with their first documented LBP diagnoses from 2012 through 2024. Exclusion criteria removed patients aged <18 years due to biological differences, patients who received spinal surgery or paraspinal injections because procedures often target specific pathological conditions, and patients with diagnoses known to cause or confound LBP such as trauma, infection, or cancer. Stratified analyses assessed patients diagnosed in the EDs and general practice settings independently.

**Figure 1.**
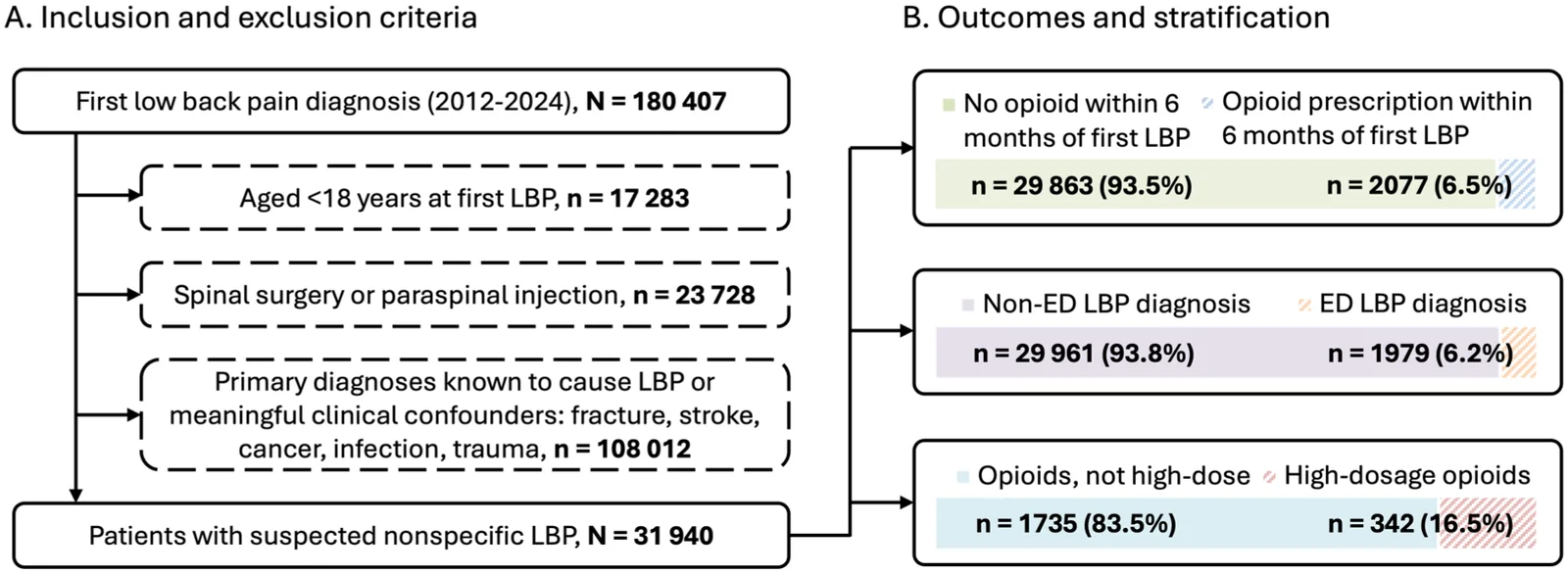
Cohort selection for patients with new-onset nonspecific low back pain (LBP), University of California, San Francisco, 2012-2024. Flowchart depicting the inclusion and exclusion criteria to identify the study cohort. Inclusion criteria identified patients with first-time presentation of LBP during the study period. Exclusion criteria were applied simultaneously to the initial cohort, removing pediatric patients, those with spinal procedures targeting specific pathology, and those with diagnoses known to confound or cause LBP. Patients meeting none of the exclusion criteria comprised the final study cohort with suspected new-onset nonspecific LBP. The patient-level outcome was opioid prescription within 6 months of first LBP diagnosis. Secondary analyses stratified patients by those with LBP diagnoses in the ED. High-dose opioid prescriptions were those exceeding 50 morphine milligram equivalents per day. Abbreviation: ED, emergency department.

The patient-level outcome was any opioid prescription within 6 months of first LBP diagnosis. We calculated prescription rates (outcome) for each quarter (effector) as the proportion of patients with an opioid prescription and first LBP diagnosis among all those with a first LBP diagnosis (quarter [Q] 1: January–March; Q2: April–June; Q3: July–September; Q4: October–December). We selected quarterly aggregation to balance temporal granularity with adequate sample sizes per time bin.

The secondary outcome assessed temporal changes in high-dose prescribing. Among patients prescribed opioids (n = 2077), daily MME doses were calculated using medication name, dosage, route, frequency, and standardized conversion factors from the National Institutes of Health HEAL (Helping to End Addiction Long-term) Initiative.^21,22^ We classified prescriptions exceeding 50 MME per day as high dose (n = 342), defined by evidence of diminished analgesic improvements in pain intensity or functional outcomes relative to risk.^11^ We used annual rather than quarterly aggregation because of high quarterly volatility (coefficient of variation, 40.0% quarterly vs 23.0% annually) that could mask intervention effects.^19,23^

### Statistical Analysis

#### Change point analysis for prescription rate inflection points

We performed change point analysis to identify periods with statistically distinct shifts in prescribing practices using quarterly prescription rates. We investigated 2 approaches to address methodological differences in model certainty and the optimal number of inflection points. We hypothesized the presence of 1 to 2 change points corresponding to major policy releases (2016 and 2022 CDC clinical guidelines), potentially delineating prepolicy, post–first guideline, and post–second guideline periods. For change point detection, we selected dynamic programming with a radial basis function (RBF) cost function because it guarantees a global solution through exhaustive search.^24^ This approach detected exactly 2 change points, consistent with our hypothesis of distinct prescribing shifts across various policy changes. For sensitivity analysis, we applied the pruned exact linear time (PELT) algorithm^25^ using an identical RBF cost with a minimum segment size of 8 quarters. Unlike dynamic programming, PELT does not require prespecification of the number of change points, instead operating by pruning candidate points.^25^ To determine the number of change points, we swept a logarithmic range of penalty values from 0.1 to 100 and applied the elbow method.

#### Interrupted time series analysis of opioid prescription rates

We performed interrupted time series analysis^19,20^ using Wagner’s segmented logistic regression to assess changes in opioid prescription rates during 3 distinct time segments. We defined time intervals between the previously identified change points 2015 Q2 and 2018 Q4 to build upon our hypothesis that change points indicate statistically significant shifts in clinical practice amid multiple opioid policy changes. The models included terms for baseline trend, level changes (β_2_, β_4_), and slope changes (β_3_, β_5_) at identified cutoff points, enabling estimation of both immediate and gradual effects as odds ratios (ORs) (OR_i_ = exp[β_i_]).^26^ Sine and cosine terms modeled seasonal variation to capture quarterly cyclicity. We estimated models using Python statsmodels generalized linear models with a binomial distribution and logit link. For comparison, we also fit a second model excluding seasonal terms.

$$\begin{array}{c} logit(p)=\beta_{0}+\beta_{1}quarters\_since\_2012Q1\\ +\beta_{2}indicator\_post\_2015Q2\\ +\beta_{3}quarters\_since\_2015Q2\\ +\;\;\beta_{4}indicator\_post\_2018Q4\\ +\beta_{5}quarters\_since\_2018Q4\\ +\beta_{6}sin_{4}(t)+\beta_{7}cos_{4}(t) \end{array}$$

Post hoc assessments ensured the model adequately captured temporal patterns and did not violate key assumptions that suggest poor model fit, biased significance tests, and biased SEs. To compare the effect of seasonality terms, we selected the best-fit model based on the lowest Akaike information criterion (AIC),^27^ which captures model fit by variance explained and complexity by penalizing the number of parameters. We checked for residual autocorrelation using the Durbin–Watson statistic (*d*),^28^ as well as autocorrelation function (ACF, with itself) and partial autocorrelation function (PACF, without previous lag effects) plots of residuals with up to 24 quarters of lag, to identify potential misspecification or unaccounted for seasonal structure.^29,30^ We ran Wald tests of regression coefficients to determine *P* values, with statistical significance set at *P* < .05.

We performed all analyses in 2025 using Python version 3.9 (Python Software Foundation) with the packages scikit-learn version 1.1.3, statsmodels version 0.13.5, and ruptures version 1.1.5.

## Results

Among 31 940 patients with ns-LBP identified during 2012-2024, a total of 2077 patients (6.5%) received opioid prescriptions within 6 months of diagnosis (Figure 1). Cohort demographic characteristics showed a mean (SD) age of 51.0 (17.6) years, 45.3% male, and 53.7% White (Table 1).

**Table 1.** Demographic Characteristics of Patients With New-Onset Nonspecific Low Back Pain (LBP), by Opioid Prescription Status and Care Setting, University of California, San Francisco, 2012-2024.^a^

Characteristic	All patients (N = 31 940)	Patients with opioid prescription^b^ (n = 2077)	Patients with ED LBP diagnosis (n = 1979)	Patients with ED LBP diagnosis and opioid prescription (n = 326)	Patients with high-dose^c^ opioid prescription (n = 342)
Age at first LBP diagnosis, mean (SD), y	51.0 (17.6)	50.2 (16.2)	45.9 (16.7)	46.6 (15.6)	53.5 (15.1)
Sex					
Male, no. (%)	14 467 (45.3)	891 (42.9)	957 (48.4)	168 (51.5)	155 (45.3)
Female, no. (%)	17 473 (54.7)	1186 (57.1)	1022 (51.6)	158 (48.5)	187 (54.7)
Obesity,^d^ no. (%)	1675 (5.2)	180 (8.7)	108 (5.5)	25 (7.7)	33 (9.6)
Race and ethnicity, no. (%)					
Asian	4042 (12.7)	197 (9.5)	245 (12.4)	40 (12.3)	31 (7.6)
Black	1991 (6.2)	174 (8.4)	346 (17.4)	44 (13.4)	30 (8.8)
Latino/Hispanic	4549 (14.2)	330 (15.9)	432 (21.8)	56 (17.2)	51 (14.9)
White	17 155 (53.7)	1157 (55.7)	671 (34.0)	146 (44.8)	204 (59.6)
Other^e^	4203 (13.2)	219 (10.5)	285 (14.4)	40 (12.3)	26 (9.1)

Abbreviation: ED, emergency department.

a Data source: Deidentified electronic health records from the University of California, San Francisco Information Commons, for patients with LBP, 2012-2024.

b Opioid prescription defined as any opioid analgesic prescribed within 6 months of first LBP diagnosis.

c High-dose opioid prescription defined as exceeding 50 morphine milligram equivalents per day.

d Obesity defined as body mass index >30 kg/m^2^ per Centers for Disease Control and Prevention classification.^31^

e “Other” category includes patients identified in the electronic health record as multiracial/ethnic, American Indian/Alaska Native, or Native Hawaiian/Other Pacific Islander, or who declined to answer.

### Change Point Detection

The dynamic programming (Dynp) algorithm, constrained to 2 change points, identified major shifts in opioid prescribing at 2015 Q2 and 2018 Q4 (Figure 2). The PELT algorithm identified a single change point at 2018 Q2 via the elbow of the penalty curve or when constrained to 2 change points at 2014 Q3 and 2018 Q2. Both algorithms converged on a transition near 2018, while the earlier transition date varied between the Dynp and PELT algorithms by 1 year.

**Figure 2.**
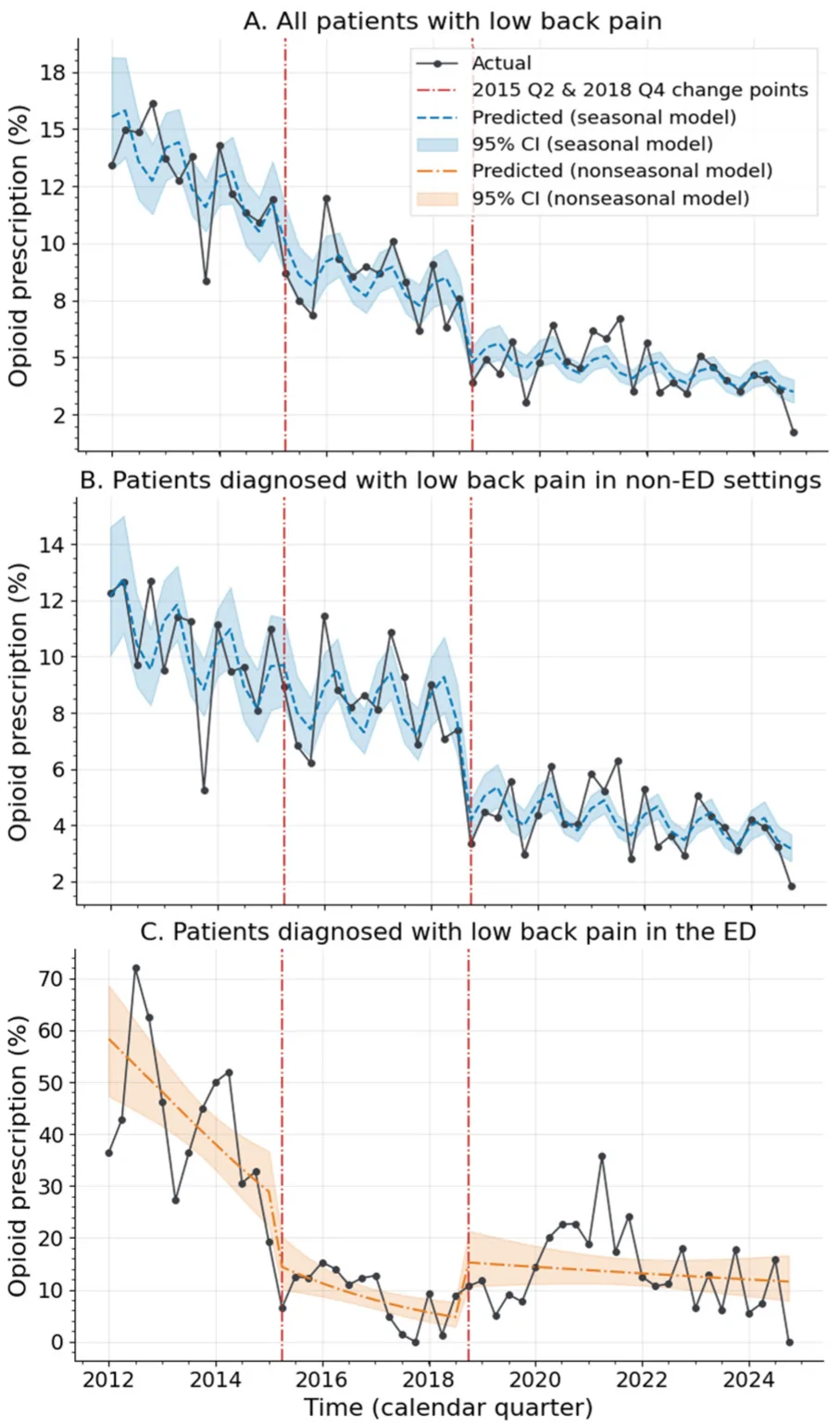
Quarterly opioid prescription rates for patients with new-onset nonspecific low back pain (LBP) and interrupted time series model fit. We identified patients with low back pain during 2012-2024 using deidentified electronic health records from the University of California, San Francisco Information Commons. Quarters were defined as Q1: January–March; Q2: April–June; Q3: July–September; Q4: October–December. All patients with LBP (n = 31 940) (A). Patients diagnosed in non-ED settings (n = 29 961) (B). Patients diagnosed in the ED (n = 1979) (C). Observed quarterly opioid prescription rates (solid line), detected change points at 2015 Q2 and 2018 Q4 (vertical lines), best fitted interrupted time series logistic regression models (dashed line) with seasonal adjustments (A, B) and without seasonal adjustment (C), and regression model 95% CIs (shaded regions). The panels use different *y*-axis scales to better show the level changes and trend modifications. Abbreviations: ED, emergency department; Q, quarter.

### Temporal Trends in Opioid Prescription Rate

The segmented logistic regression model with seasonality demonstrated better fit (ΔAIC = 7.99) than the model without seasonality, with no evidence of autocorrelation (*d* = 2.00) and adequate residual patterns indicating appropriate fit (model fit statistics in eTable 1 in the Supplement).

Interrupted time series trends revealed a consistent quarterly decline in opioid prescription odds throughout the study period (OR_1_; *P* = .024), corresponding to a 2.7% decrease per quarter (Figure 2A, Table 2 [model with seasonality terms], and eTable 1 [model without seasonality terms] in the Supplement).

**Table 2.** Odds Ratios From Interrupted Time Series Logistic Regression Model for Opioid Prescribing Among Patients With New-Onset Nonspecific Low Back Pain, University of California, San Francisco, 2012-2024 (N = 31 940).^a^

Variable	Time frame^b^	Comparison (reference vs event)^b^	OR (95% CI)^c^	*P* value^c^
$OR_{0}$	All	No opioid vs opioid	0.175 (0.147-0.208)	<.001
$OR_{1}$	Pre-2015 Q2	Opioid at quarter t vs t + 1	0.973 (0.950-0.996)	.02
$OR_{2}$	At 2015 Q2	Opioid pre- vs post-2015 Q2	0.818 (0.646-1.036)	.10
$OR_{3}$	Post-2015 Q2	Opioid at quarter t vs t + 1	1.012 (0.981-1.044)	.45
$OR_{4}$	At 2018 Q4	Opioid pre- vs post-2018 Q4	0.679 (0.542-0.850)	.001
$OR_{5}$	Post-2018 Q4	Opioid at quarter t vs t + 1	1.001 (0.979-1.024)	.90
$OR_{6}$	All	Opioid at sine peak vs trough	1.104 (1.037-1.176)	.002
$OR_{7}$	All	Opioid at cosine peak vs trough	1.053 (0.987-1.123)	.12

Abbreviations: OR, odds ratio; Q, quarter; t, timepoint.

a Data source: Deidentified electronic health records from the University of California, San Francisco Information Commons, for patients with low back pain, 2012-2024.

b Quarters were defined as Q1: January–March; Q2: April–June; Q3: July–September; Q4: October–December.

c *P* values and 95% CIs were calculated using the Wald test. Significance was set at *P* < .05.

At the 2015 Q2 transition, we observed an immediate 18.2% reduction in opioid prescription odds, although the reduction did not reach significance (OR_2_; *P* = .10). In contrast, the 2018 Q4 transition demonstrated a significant 32.1% immediate reduction (OR_4_; *P* = .001). Post-transition slope changes were nonsignificant in both periods (OR_3_; *P* = .45; OR_5_; *P* = .90), thereby indicating that the underlying quarterly decline remained stable throughout the 48-quarter study period. Thus, the decline specified by OR_1_ corresponds to an estimated 72% cumulative reduction in opioid prescription odds from baseline (100 – 100 × 0.973^48^ = 72%).

We detected minor seasonal variation (OR_6_; *P* = .002), with 10.4% higher prescription odds at Q4 peak versus Q2 trough periods.

### Stratification by ED Diagnosis Setting

Stratified analyses revealed divergent prescribing trajectories between ED and non-ED settings (Table 3; Figure 2B and C). Of 31 940 patients, 1979 (6.2%) received their LBP diagnosis in the ED, among whom 326 (16.5%) were prescribed opioids. In comparison, 1751 of 29 945 non–ED-diagnosed patients (5.8%) were prescribed opioids. For non-ED patients, the best-fit model had seasonality terms (ΔAIC = 6.11; *d* = 2.02), while for ED patients, the best-fit model did not include seasonality (ΔAIC = −1.29; *d* = 2.00).

**Table 3.** Odds Ratios From Interrupted Time Series Logistic Regression Model for Opioid Prescribing Among Patients With New-Onset Nonspecific Low Back Pain (LBP), Stratified by Care Setting (Non-ED: n = 29 961; ED: n = 1979), University of California, San Francisco, 2012-2024.^a^

			Within strata of non-ED LBP diagnosis		Within strata of ED LBP diagnosis	
Variable	Time frame^b^	Comparison (reference vs event)^b^	OR (95% CI)^c^	*P* value^c^	OR (95% CI)^c^	*P* value^c^
$OR_{0}$	All	No opioid vs opioid	0.130 (0.106-0.159)	<.001	1.400 (0.897-2.186)	.14
$OR_{1}$	Pre-2015 Q2	Opioid at quarter t vs t + 1	0.979 (0.952-1.006)	.12	0.902 (0.852-0.955)	<.001
$OR_{2}$	At 2015 Q2	Opioid pre- vs post-2015 Q2	0.949 (0.728-1.237)	.70	0.458 (0.258-0.812)	.008
$OR_{3}$	Post-2015 Q2	Opioid at quarter t vs t + 1	1.017 (0.983-1.053)	.33	1.009 (0.930-1.096)	.83
$OR_{4}$	At 2018 Q4	Opioid pre- vs post-2018 Q4	0.572 (0.451-0.725)	<.001	3.986 (1.994-7.973)	<.001
$OR_{5}$	Post-2018 Q4	Opioid at quarter t vs t + 1	0.992 (0.969-1.016)	.52	1.084 (1.014-1.160)	.02
$OR_{6}$	All	Opioid at sine peak vs trough	1.153 (1.078-1.235)	<.001	NA	NA
$OR_{7}$	All	Opioid at cosine peak vs trough	1.065 (0.993-1.142)	.08	NA	NA

Abbreviations: ED, emergency department; NA, not applicable; OR, odds ratio; Q, quarter; t, timepoint.

a Data source: Deidentified electronic health records from the University of California, San Francisco Information Commons, for patients with LBP, 2012-2024.

b Quarters were defined as Q1: January–March; Q2: April–June; Q3: July–September; Q4: October–December.

c *P* values and 95% CIs were calculated using the Wald test. Significance was set at *P* < .05.

ED-diagnosed patients had higher baseline prescription odds (OR_0_ = 1.400; *P* = .14) than non-ED patients (OR_0_ = 0.130; *P* < .001), although the ED estimate was not statistically significant. Pre–2015 Q2, ED prescribing odds declined significantly at 9.8% per quarter (OR_1_; *P* < .001), while non-ED prescribing odds had a nonsignificant decline (OR_1_; *P* = .12).

Prescription rates diverged between ED and non-ED settings at each transition point. At 2015 Q2, ED-diagnosed patients had a significant 54.2% immediate reduction in prescription odds (OR_2_; *P* = .008), whereas non-ED patients had no significant change (OR_2_; *P* = .70). At 2018 Q4, the pattern reversed as non-ED patients demonstrated a significant 42.8% reduction (OR_4_; *P* < .001), consistent with the overall model, while the ED stratum had a significant 4.0% level change in the opposite direction (OR_4_; *P* < .001). Notably, OR_4_ reflected a shift relative to the steeply declining pre–2018 Q4 ED trajectory rather than an absolute increase. Thus, ED opioid prescription odds continued to decline from 27.4% to 12.2% to 10.6% across these periods.

Post–transition slope changes were non-significant for non-ED patients in both periods (OR_3_; *P* = .33; OR_5_; *P* = .52) and for ED patients post–2015 Q2 (OR_3_; *P* = .83). However, ED patients had a significant positive slope change during post–2018 Q4 (OR_5_ = 1.084; *P* = .02), indicating a substantial deceleration in the rate of decline, with the combined quarterly trend flattening from approximately a 9.8% to 9.0% to 1.4% odds decline per quarter. We detected seasonal variation only among non-ED patients (OR_6_; *P* < .001) and represented a 15.3% difference in prescription odds between peak and trough periods.

### Temporal Trends in High-Dose Opioid Prescription

Secondary analysis revealed no statistically significant immediate effects or trend changes in the prevalence of high-dose opioid prescriptions among ns-LBP patients with an opioid prescription, despite guideline updates that recommended doses below the 50 MME/day threshold (eTable 2 and eFigure 1 in the Supplement).

## Discussion

While previous studies have documented prescribing reductions following guideline releases,^16-18,32-36^ our study examined opioid prescribing rates for new-onset ns-LBP patients across a 12-year period spanning multiple policy interventions. Our analysis identified distinct periods of prescription shifts that aligned with anticipatory adoption, gradual transition, and immediate regulatory response.

### Sustained Reductions in ns-LBP Opioid Prescribing

Opioid prescribing odds declined 2.7% per quarter across the full study period, yielding a cumulative 72% reduction. The steady linear decline indicates that prescribing odds decreased at a consistent rate across all 3 policy segments, consistent with overarching policy messaging, despite differing enforcements. This uniform trajectory reflected accumulating empirical evidence and evolving professional consensus supporting cautionary opioid use for ns-LBP rather than any single policy event. These changes are consistent with the limited analgesic efficacy of opioids for nonspecific pain relative to placebo and the documented misuse risk doubling to 20% during a 1-year period,^3,8,37,38^ although we did not assess pain outcomes in this study.

In our study, UCSF’s 10.8% annual ns-LBP prescription reduction exceeded California’s 5.9% annual decline in short-duration (<3 d) general opioid prescribing across all indications and diverged from a 3.1% annual increase in long-duration (≥30 d) prescriptions.^39^ Although these comparisons span different patient populations and indications, this contrast suggests that condition-specific prescribing for ns-LBP, an evidence-poor indication, was more responsive to guideline pressure than aggregate prescribing, where competing patient needs likely moderated policy effects.

### Implementation Mechanism Determines Adoption Timeline

Change point analysis identified transition points that aligned with the 2016 CDC guideline and 2018 California Prescription Drug Monitoring Programs implementation, despite using solely observed data without policy priors. The gradual, nonsignificant level change at 2015 Q2 versus the abrupt, significant reduction at 2018 Q4 is consistent with distinct clinical adoption pathways. These transitions suggest that voluntary guidelines operate through knowledge diffusion with sequential adoption, whereas mandatory regulations operate through compliance mechanisms that bypass individual adoption decisions.

The 2015 Q2 transition preceded the official 2016 CDC chronic pain guideline by approximately 1 year, coinciding with the draft circulation for public comment and heightened media discourse on opioid risks.^40^ This anticipatory effect aligns with policy diffusion theory mechanisms (learning, emulation, coercion, and social contagion),^41^ where early adopters respond to emerging evidence before formal recommendations, with physicians in the highest medical knowledge quartile reducing LBP opioid prescribing 4.6% more than colleagues in the lowest knowledge quartile.^16^ The subsequent 36-month transition period before the next inflection captures the time required for broader translation including health care provider training programs, institutional policy updates, and care pathway modifications.^33,42^ Voluntary guidelines face inherent adoption barriers, including resistance to change, increased clinical time demands, and interdisciplinary coordination requirements^12,18^ that may explain the gradual transition despite strong underlying evidence.

The 2018 Q4 transition demonstrates a fundamentally different policy mechanism. The immediate level decline, which is consistent with immediate general opioid prescription changes,^43^ coincided with 2 concurrent California mandates that imposed new procedural requirements at the time of prescribing. Assembly Bill No. 2760 (September 10, 2018) required naloxone coprescribing and overdose education as preventive measures,^12^ and CURES Mandatory Consultation (October 2, 2018) required controlled-substance history review before prescribing Schedule II-IV opioids.^13,14^ Unlike guidelines that require clinicians’ decision to engage, policymakers designed these mandates to alter the clinical prescribing environment itself. Findings are consistent with evidence that state regulations reduced new opioid prescriptions while federal guidelines primarily affected opioid continuation.^44^ The distinction between modifying behavior individually versus modifying the choice architecture has direct implications for policy design. When rapid adoption is required, mandatory regulations that restructure clinical workflows are more effective than informational interventions targeting clinician knowledge. Thus, hybrid policy approaches combining recommendations with regulatory enforcement may optimize both adoption speed and clinical acceptance.

### Department Setting–Specific Responses

ED opioid prescribing declined 26.8% overall, with the steepest reductions occurring before and immediately after the 2015 Q2 transition, consistent with national data showing LBP ED prescribing falling from 32% in 2016 to 13% in 2022.^45^ This pattern suggests that voluntary guideline efforts contributed to disproportionate, rapid impact in acute care settings where opioid prescription was most concentrated. The post–2018 Q4 plateau from 12.4% to 10.6% likely reflects a pragmatic bounded effect due to high-acuity presentations where nonopioid alternatives remain limited and prior findings of limited drug monitoring program effects in the ED.^46^ Nonsteroidal anti-inflammatory drugs are a guideline-concordant first-line opioid alternative for LBP, but their use is constrained by contraindications (eg, renal disease, anticoagulation) and ceiling effects for severe pain, limiting their ability to function as a direct replacement. ED studies report that opioids are preferentially used in patients with high back-pain scores^45,47^ but without better pain score outcome,^48,49^ increased health care use,^50,51^ and increased risk of ongoing use^51-53^ after adjustment for baseline pain. The persistence of high-dose prescribing and its substantial variability^52^ highlights the need for clinical decision support tools that promote opioid alternatives, an approach known to reduce health care use,^54-56^ in addition to the evaluation of patient-centered outcomes.

### High-Dose Prescription Rates Unaffected

Among opioids prescribed, the proportion exceeding 50 MME/day remained unchanged, even though CDC guidelines recommend nonsteroidal anti-inflammatory drugs as a first-line pharmacologic intervention and advise caution with opioid prescribing.^10^ For ns-LBP, this divergence suggests that policy interventions influenced the decision to initiate opioids but not dosage intensity among those treated. Sustained high-dose prescribing likely reflects pain management challenges given limited alternatives and difficulty aligning care with patient expectations.^3,57^

Comparisons between this study and prior work are constrained by differences in outcome definitions and study populations. Across all indications in the United States, prescribing at ≥50 MME/day and/or with concurrent benzodiazepines fell by 2.6% from 12.0% in 2011 to 9.4% in 2016,^58^ suggesting that ns-LBP prescribing intensity is less responsive to current policy approaches than prescribing for all indications. Internationally, publication of the 2013 Scottish national pain guideline coincided with an overall decline in prescribing alongside continued growth in strong-opioid prescribing (buprenorphine, diamorphine, dipipanone, fentanyl, hydromorphone, methadone, morphine, oxycodone, papaveretum, pentazocine, pethidine, and tapentadol).^34^ Scandinavian and Irish studies identified rising strong-opioid use^59,60^ with concurrent declines in average dose.^60^ Although these studies do not report standardized MME, the persistence of strong-opioid prescribing across settings suggests a shared constraint on non-opioid treatment options. For ns-LBP, further policy-related dose reductions may depend on the availability of effective alternative pain management strategies.

### Limitations

This study had several limitations. First, the single-institution design may not generalize to other health care settings, and analyses assume accurate database documentation. Second, the interrupted time series analysis identified population-level temporal shifts in prescription patterns relative to the baseline trend rather than causal effects, yet this methodology allows for policy evaluation when controls are infeasible. Third, ORs should be interpreted carefully, because percentage changes in odds are not equivalent to percentage changes in probability. Fourth, our analysis cannot distinguish between prescriber-initiated changes and shifts in patient presentation or care-seeking behavior. The COVID-19 pandemic may have influenced prescribing patterns in later study periods, although the steady quarterly trend indicates no substantial disruption to the underlying trajectory. Fifth, while we assessed opioid prescribing rates, future investigations may incorporate prescription duration,^35^ alternative treatment use,^36,57^ patient-reported pain outcomes, and subgroup analyses, such as by age,^58^ to provide additional clinical context. Subsequent investigations may link these temporal patterns with patient-level characteristics and patient-reported outcomes.

## Conclusion

Declines in opioid prescribing followed 2 distinct temporal patterns, gradual reductions preceding voluntary federal guidelines consistent with knowledge diffusion, and immediate shifts coinciding with mandatory state regulations, with effects concentrated in different clinical settings. Overall, prescribing for ns-LBP declined 72% during 12 years amid these evolving policies. Voluntary CDC guidelines coincided with anticipatory, gradual adoption beginning in 2015, concentrated in EDs where baseline prescribing was highest. Mandatory prescription monitoring requirements coincided with immediate compliance effects in 2018, predominantly in non-ED settings. However, high-dose prescribing rates persisted among initiated prescriptions, suggesting current policies altered the decision to prescribe but not prescribing intensity. These findings can inform targeted interventions matching policy design to clinical context, although whether reduced prescribing improved pain outcomes warrants further study.

## Supplemental Material

sj-docx-1-phr-10.1177_00333549261486260 – Supplemental material for Trends in Opioid Prescribing for Nonspecific Low Back Pain After CDC Guidelines and California Prescription Drug Monitoring Programs, 2012-2024 Supplemental material, sj-docx-1-phr-10.1177_00333549261486260 for Trends in Opioid Prescribing for Nonspecific Low Back Pain After CDC Guidelines and California Prescription Drug Monitoring Programs, 2012-2024 by Michelle W. Tong, Gabby B. Joseph and Sharmila Majumdar in Public Health Reports®

## Data Availability

Code is available in a public repository for transparency and reproducibility: https://github.com/michelle-tong18/temporal-trends-in-opioids.
