# Supplementary material for: Trends in Opioid Prescribing for Nonspecific Low Back Pain After CDC Guidelines and California Prescription Drug Monitoring Programs, 2012-2024
Source: Public Health Rep. 2026 Oct 25:00333549261486260. Online ahead of print. doi: 10.1177/00333549261486260 (PMC13696577; doi:10.1177/00333549261486260)
Supplement: Supplementary file 1 [file sj-docx-1-phr-10.1177_00333549261486260.docx]

**Supplemental Materials**

Table of Contents

[Supplemental Table1: Interrupted time series model fit parameters	2](#_Toc235836408)

[Supplemental Table 2: Odds ratios for ns-LBP model without seasonality	3](#_Toc235836409)

[Supplemental Table 3: Odds ratios for annual high-dosage model within strata of opioid prescribed patients	4](#_Toc235836410)

[Supplemental Figure 1: High-dose prescription rates within strata of opioid prescribed patients	5](#_Toc235836411)

[Supplemental Code Snippet 1: Patient inclusion criteria	6](#_Toc235836412)

[Supplemental Code Snippet 2: Patient LBP diagnosis names	7](#_Toc235836413)

[Supplemental Code Snippet 3: Patient exclusion criteria	14](#_Toc235836414)

# **Supplemental Table1: Interrupted time series model fit parameters**

Comparison of model fit statistics for interrupted time series logistic regression models with and without seasonality components across the overall nonspecific low back pain (ns-LBP) cohort^a^ (N = 31 940), stratified by emergency department diagnosis setting (non-ED n = 29 961; ED n = 1979), and comparing high- vs low- dose prescriptions^b^. The preferred model for each stratum was selected based on lower Akaike Information Criterion (AIC). Durbin-Watson d statistics near 2.00 indicate no autocorrelation.

| # | Patient Strata for Opioid Prescription Rate Model | Seasonality | AIC | Log-Likelihood | Pseudo R² | Durbin-Watson d | Best Model |
| --- | --- | --- | --- | --- | --- | --- | --- |
| 1 | All new-onset ns-LBP | Yes | 14931.15 | -7457.6 | 0.01409 | 2.00 | **True** |
| 2 | All new-onset ns-LBP | No | 14939.14 | -7463.6 | 0.01372 | 1.99 | False |
| 3 | Non-ED diagnosed LBP | Yes | 13032.19 | -6508.1 | 0.01082 | 2.02 | **True** |
| 4 | Non-ED diagnosed LBP | No | 13048.30 | -6518.1 | 0.01016 | 2.02 | False |
| 5 | ED diagnosed LBP | Yes | 1584.31 | -784.16 | 0.09732 | 2.00 | False |
| 6 | ED diagnosed LBP | No | 1583.02 | -785.51 | 0.09608 | 2.00 | **True** |
| 7 | Any opioid (dose model) | Yes | 1822.99 | -903.49 | 0.006407 | 2.03 | False |
| 8 | Any opioid (dose model) | No | 1819.27 | -903.63 | 0.006268 | 2.03 | **True** |

Abbreviation: ED, emergency department; AIC, Akaike Information Criterion.

^a^ Data source: Deidentified electronic health records from the University of California, San Francisco Information Commons, for patients with LBP, 2012-2024.

^b^ High-dose opioid prescription defined as exceeding 50 morphine milligram equivalents per day.

# **Supplemental Table 2: Odds ratios for ns-LBP model without seasonality**

Interrupted time series logistic regression models parameters excluding seasonality for patients with ns-LBP^a^. Odds ratios (ORs) reveal a significant 3.0% quarterly decline in the odds of an opioid prescription for ns-LBP, alongside an abrupt decline amounting to 32.2% reduction in odds in 2018Q4 indicated by a significant level shift.

| Variable | Timeframe | Comparison (Reference vs Event) | OR (95% CI)^b^ | P-value^b^ |
| --- | --- | --- | --- | --- |
| $\boldsymbol{OR}_{\boldsymbol{0}}$ | All | No opioid vs. opioid | 0.178 (0.149 - 0.212) | < .001 |
| $\boldsymbol{OR}_{\boldsymbol{1}}$ | Pre-2015Q2 | Opioid at quarter^c^ t vs. t+1 | 0.970 (0.948 - 0.994) | .012 |
| $\boldsymbol{OR}_{\boldsymbol{2}}$ | At 2015Q2 | Opioid pre- vs. post- 2015Q2 | 0.837 (0.661 - 1.059) | .137 |
| $\boldsymbol{OR}_{\boldsymbol{3}}$ | Post-2015Q2 | Opioid at quarter t vs. t+1 | 1.015 (0.984 - 1.047) | .349 |
| $\boldsymbol{OR}_{\boldsymbol{4}}$ | At 2018Q4 | Opioid pre- vs. post- 2018Q4 | 0.673 (0.538 - 0.843) | .001 |
| $\boldsymbol{OR}_{\boldsymbol{5}}$ | Post-2018Q4 | Opioid at quarter t vs.t+1 | 1.002 (0.979 - 1.024) | .875 |

Abbreviation: OR, odds ratio; CI, confidence interval; Q, quarter ^c^; t, timepoint.

^a^ Data source: Deidentified electronic health records from the University of California, San Francisco Information Commons, for patients with low back pain, 2012-2024.

^b^ *P* values and 95% CIs were calculated using Wald test. Significance was set at P < .05.

^c^ Quarters were defined as Q1: January–March; Q2: April–June; Q3: July–September; Q4: October–December.

# **Supplemental Table 3: Odds ratios for annual high-dosage model within strata of opioid prescribed patients**

Odds ratios (ORs) derived from interrupted time series logistic regression evaluation high-dosage opioid prescription rates among ns-LBP patients with an opioid prescription^a^. For patients who received an opioid prescription, the odds of high-dosage^b^ was substantially lower than low-dosages prior to guideline updates (OR_0_ 0.26). There was no evidence of meaningful year-to-year change in high-dose prescribing over the study period (OR₁ 1.01), and neither Center for Disease Control and Prevention nor California policy changes were associated with significant immediate level changes or subsequent trend shifts (OR₂–OR₅, all p>0.05). Overall, percent prevalence of high-dose prescribing for new-onset ns-LBP remained consistent with minimal change over time and no detectable impact of guideline implementation.

| Variable | Timeframe | Comparison (Reference vs Event) | OR (95% CI)^c^ | P-value^c^ |
| --- | --- | --- | --- | --- |
| $\boldsymbol{OR}_{\boldsymbol{0}}$ | All | Low vs. high dose opioid | 0.264 (0.187 - 0.374) | < .001 |
| $\boldsymbol{OR}_{\boldsymbol{1}}$ | Pre-2015 | High-dose at year t vs. t+1 | 1.01 (0.783 - 1.30) | .946 |
| $\boldsymbol{OR}_{\boldsymbol{2}}$ | At 2015 | High-dose pre- vs. post- 2015 | 0.886 (0.473 - 1.59) | .705 |
| $\boldsymbol{OR}_{\boldsymbol{3}}$ | Post-2015 | High-dose at year t vs. t+1 | 0.963 (0.704 - 1.32) | .809 |
| $\boldsymbol{OR}_{\boldsymbol{4}}$ | At 2019 | High-dose pre- vs. post- 2019 | 0.763 (0.403 - 1.45) | .407 |
| $\boldsymbol{OR}_{\boldsymbol{5}}$ | Post-2019 | High-dose at year t vs.t+1 | 1.03 (0.826 - 1.28) | .803 |

Abbreviation: OR, odds ratio; CI, confidence interval; t, timepoint.

^a^ Data source: Deidentified electronic health records from the University of California, San Francisco Information Commons, for patients with low back pain, 2012-2024.

^b^ High-dose opioid prescription defined as exceeding 50 morphine milligram equivalents per day.

^c^ *P* values and 95% CIs were calculated using Wald test. Significance was set at P < .05.

# **Supplemental Figure 1: High-dose prescription rates within strata of opioid prescribed patients**

Plot of interrupted time series logistic regression models fit for the percentage prevalence of high-dosage opioid prescription relative to all opioid prescriptions for ns-LBP patients^a^. We identified patients with lower back pain during 2012-2024 from the University of California, San Francisco (UCSF) deidentified electronic health records “Information Commons”. A scatterplot (dots) displays percentage of high-dosage (exceeding 50 morphine milligram equivalents per day) opioid prescriptions annually from 2012 to 2024. Vertical lines separate three distinct time intervals between 2015 and 2019 from change point analysis. Logistic regression model fit and 95% confidence intervals are visualized by trend lines for the best-fit model (dashed line and shaded region). The best-fit model did not detect significant changes in high-dosage opioid prescription prevalence over time.

# **Supplemental Code Snippet 1: Patient inclusion criteria**

SQL queries of the de-identified electronic health record.

```
diagnosis_query1 = f'''
/*
Description: Select patients with LBP
*/
SELECT DISTINCT
  diagnosiskey,
  diagnosisname,
  type,
  value
FROM diagnosisterminologydim
WHERE
  (
    (
      LOWER(diagnosisname) LIKE '%back%pain%'
      AND
      (
      LOWER(diagnosisname) LIKE '%lumb%'
      OR LOWER(diagnosisname) LIKE '%low%'
      )
    )
    OR LOWER(diagnosisname) LIKE '%lbp%'
    OR
    (
      value LIKE 'M51.1'
      OR value LIKE 'M54.4'
      OR value LIKE 'M54.9'
    )
  )
ORDER BY type, value
'''

diagnosis_query2 = f'''
/*
Description: Select patients with LBP
*/
SELECT DISTINCT
  patientdurablekey,
  diagnosisname,
  startdatekey,
  startdatekeyvalue
FROM
  diagnosiseventfact
  WHERE
    diagnosiskey IN {tuple(LBP_key_query1_list)}
    AND
    startdatekey > 0

  ORDER BY
    startdatekey,
    patientdurablekey
'''
```

# **Supplemental Code Snippet 2: Patient LBP diagnosis names**

269 distinct LBP diagnosis codes across ICD-10-CM, ICD-9-CM, and SNOMED CT; and 487 distinct LBP diagnosis names that matched the inclusion criteria from **diagnosis_query1** in *Supplemental Code Snippet 1*.

**ICD-10-CM:** G89.11; G89.29; H35.52; M51.1; M51.26; M51.360; M51.362; M51.369; M51.370; M51.372; M51.379; M51.9; M53.9; M54.10; M54.16; M54.4; M54.40; M54.41; M54.42; M54.5; M54.50; M54.51; M54.59; M54.6; M54.9; M79.604; M79.605; M79.606; O26.891; O26.892; O26.893; O26.899; Z15.89; Z82.69; Z87.39

**ICD-9-CM:** 338.11; 338.19; 338.29; 362.74; 646.80; 646.83; 722.10; 724.1; 724.2; 724.3; 724.4; 724.5; 724.6; 739.3; 846.0; 846.9; 847.2; 847.3; 847.4; 959.19; V13.59; V17.89; V84.89

**SNOMED CT:** 102957003; 103016004; 105969002; 10601006; 106028002; 106147001; 106221001; 11131000119108; 1145238004; 1145241008; 1156802000; 116225000; 116306000; 116312005; 11679003; 118185001; 118235002; 118934005; 118937003; 118940003; 118948005; 118952005; 118953000; 118954006; 119414006; 123798002; 12584003; 1269101009; 127325009; 1279550006; 128121009; 128127008; 128135006; 128196005; 128237006; 128268005; 128536001; 128605003; 129139009; 130731000119100; 13211000119109; 134407002; 138875005; 15633361000119103; 15634391000119107; 15634511000119108; 15968741000119100; 16026551000119108; 161570007; 161891005; 161892003; 16206701000119101; 16269008; 16638401000119101; 16839401000119104; 173300003; 1899006; 19660004; 198609003; 202708005; 202794004; 22253000; 23056005; 232053004; 239880009; 243796009; 246595001; 246605000; 246915008; 247366003; 247368002; 248402002; 248982007; 25416002; 26538006; 267982002; 274663001; 27624003; 276435006; 278860009; 278862001; 279001004; 279038004; 279039007; 279040009; 279055000; 279062009; 279069000; 280133005; 280134004; 280136002; 287047008; 287048003; 28835009; 289205009; 291921000119100; 291931000119102; 292501000119108; 292511000119106; 292521000119104; 292531000119101; 29555009; 298364001; 298379008; 298385001; 298671000; 29930001000004103; 300957005; 301366005; 302226006; 302292003; 302293008; 312225001; 312771007; 313501000119105; 314407005; 314642004; 316821000119105; 316961000119107; 320281000119104; 320291000119101; 32895009; 33308003; 362965005; 362975008; 363055007; 363059001; 363137000; 363343008; 36427004; 366323009; 371405004; 371409005; 372109003; 373621006; 386033004; 38781000087100; 399194009; 399269003; 399858007; 399986003; 404684003; 406122000; 410730009; 413350009; 414252009; 41587001; 416471007; 417662000; 41799005; 419003001; 426469008; 42658009; 427653003; 429976008; 431521000; 441742003; 443700006; 451421000124102; 451431000124104; 48926009; 50927007; 52585001; 55446002; 57177007; 57630001; 58184002; 59466002; 608830002; 608832005; 60937000; 609496007; 609600000; 609612001; 609619005; 609620004; 609623002; 609624008; 609625009; 609626005; 616839841000119101; 62585004; 631000119102; 64572001; 699697007; 699699005; 700012005; 713314006; 715057009; 72274001; 73589001; 737305006; 762305005; 762452003; 762591005; 77547008; 782964007; 78514002; 785875003; 822987005; 822988000; 82423001; 85995004; 88230002; 8847002; 90834002; 9121000119106; 91957002; 928000; 95695004; 9972008

**Diagnosis names:** Chronic right-sided low back pain; Chronic low back pain with right-sided sciatica; Chronic right-sided low back pain with sciatica; Thoracic, thoracolumbar and lumbosacral intervertebral disc disorders with radiculopathy; Lumbago with sciatica; Chronic low back pain with right-sided sciatica, unspecified back pain laterality; Bilateral low back pain with bilateral sciatica; Right low back pain, unspecified chronicity, with sciatica presence unspecified; Bilateral low back pain, with sciatica presence unspecified; Acute bilateral low back pain, with sciatica presence unspecified; Chronic left-sided low back pain without sciatica; Acute right-sided low back pain, unspecified whether sciatica present; Chronic right-sided low back pain, unspecified whether sciatica present; Acute low back pain without sciatica; Nonspecific low back pain; Low back pain, unspecified back pain laterality, unspecified chronicity, unspecified whether sciatica present; Chronic left-sided low back pain, unspecified whether sciatica present; Intermittent low back pain; Moderate back pain; Back pain associated with peripheral numbness; Chronic midline back pain, unspecified back location; Back pain during pregnancy in first trimester; Back pain in pregnancy, first trimester; Postoperative back pain; Bilateral back pain, unspecified back location, unspecified chronicity; Acute back pain; Chronic left-sided back pain, unspecified back location; Back pain during pregnancy in second trimester; Pain in back; Spine pain, multilevel; Pregnancy related back pain in first trimester, antepartum; Acute bilateral back pain; Back pain with radiation; Upper back pain on left side; Pain, upper back; Pain of mid back; Right-sided back pain; Back pain affecting pregnancy in second trimester; Atypical back pain; Multilevel spine pain; Intractable back pain; Pregnancy related back pain, antepartum; Chronic back pain greater than 3 months duration; Back pain, unspecified location; Mid back pain; Acute right-sided back pain; Bilateral back pain; Pain of facet joint; Pregnancy related back pain, antepartum, unspecified trimester; Acute midline back pain; Back pain affecting pregnancy; Chronic back pain, unspecified back location, unspecified back pain laterality; Notalgia; Chronic bilateral back pain; Back pain; Pregnancy related back pain, antepartum, first trimester; Back pain as manifestation of blood transfusion reaction, subsequent encounter; Spinal column pain; Back pain in pregnancy; Back pain, acute; Costovertebral angle tenderness; Tenderness of back; Spine pain; Left-sided back pain, unspecified back location, unspecified chronicity; Pregnancy with back pain, antepartum; Acute bilateral back pain, unspecified back location; Back pain complicating pregnancy; Exacerbation of backache; Back pain as manifestation of blood transfusion reaction, sequela; Back pain, unspecified back location, unspecified back pain laterality, unspecified chronicity; Acute back pain, unspecified back location, unspecified back pain laterality; Back pain worse on sneezing; Dorsodynia; Back arthralgia; Back pain complicating pregnancy in second trimester; Back pain complicating pregnancy, unspecified trimester; Chronic right-sided back pain, unspecified back location; Back pain, unspecified back pain laterality, unspecified location; Trigger point with back pain; Pain of back and lower extremity; Vertebral column pain; Disabling back pain; Pain of costovertebral angle; Pain of vertebral joint; Back pain as manifestation of blood transfusion reaction; Musculoskeletal back pain; Spinal pain; Left-sided back pain; Chronic back pain; Acute on chronic back pain; Chronic high back pain; Backache with radiation; Acute back pain less than 4 weeks duration; Pregnancy related back pain, antepartum, third trimester; Backache, unspecified; Dorsalgia; Acute left-sided back pain, unspecified back location; Backache worse with sneezing; Acute mid back pain; Back pain due to injury; Bilateral back pain, unspecified location; Back pain, chronic; Tenderness over spine; Backache without radiation; Back pain during pregnancy; Pain of back and left lower extremity; Back pain with rapidly progressive neurological deficit; Chronic neck and back pain; Back pain with sciatica; Acute right-sided back pain, unspecified back location; Chronic mid back pain; CVA tenderness; Dorsal back pain; Back pain complicating pregnancy in third trimester; Acute upper back pain; Mid back pain, chronic; Upper back pain; Non-traumatic mid back pain; Back pain in pregnancy, unspecified trimester; Backache symptom; Discomfort of back; Pregnancy related back pain, antepartum, second trimester; Severe back pain; Arthralgia of back; Dorsalgia of multiple sites in spine; Backache; Acute midline back pain, unspecified back location; Upper back pain, chronic; Mid back pain on left side; Back pain complicating pregnancy in first trimester; Subacute back pain; Back pain with risk for osteoporosis; Costovertebral angle pain; Chronic pain after spinal surgery; Chronic left-sided back pain; Dorsal pain; Back pain without radiation; Chronic bilateral back pain, unspecified back location; Pain of back and right lower extremity; Back pain with history of spinal surgery; Back pain complicating pregnancy, first trimester; Pain in spinal column; Back ache; Back pain affecting pregnancy in first trimester; Chronic right-sided back pain; Back pain in pregnancy, third trimester; Back pain in pregnancy, second trimester; Back pain without sciatica; Pain radiating to back; Back pain affecting pregnancy, antepartum; Exacerbation of chronic back pain; Mid-back pain, acute; Mid back pain on right side; Back pain as manifestation of blood transfusion reaction, initial encounter; Back pain during pregnancy in third trimester; Back pain, subacute; Back pain complicating pregnancy, second trimester; Chronic upper back pain; Back pain without radiculopathy; Back pain affecting pregnancy in third trimester; Pregnancy related back pain in third trimester, antepartum; Upper back pain on right side; Pain in right paraspinal region; Left-sided back pain, unspecified location; Pain in vertebral column; Chronic midline back pain; Back pain with sciatica, unspecified laterality; Back pain complicating pregnancy, third trimester; Right-sided back pain, unspecified back location, unspecified chronicity; Mechanical back pain; Radiating back pain; Acute left-sided back pain; Mild back pain; Dorsalgia, unspecified; Pregnancy related back pain in second trimester, antepartum; Low back pain radiating down leg; Low back pain during pregnancy, second trimester; Hx of low back pain; Chronic midline low back pain, with sciatica presence unspecified; Left-sided low back pain without sciatica; Chronic midline low back pain with sciatica; Other low back pain; Low back pain potentially associated with spinal stenosis; Chronic bilateral low back pain with left-sided sciatica; LBP radiating to right leg; Lumbosacral back pain; Severe low back pain; Low back pain, unspecified laterality, with sciatica presence unspecified, sciatica laterality unspecified; Chronic low back pain, unspecified back pain laterality, with sciatica presence unspecified; Low back pain with sciatica, unspecified laterality; Midline low back pain with sciatica, sciatica laterality unspecified; Low back pain with neuralgia of right sciatic nerve; Chronic left-sided low back pain with sciatica, sciatica laterality unspecified; Low back pain with radiation, unspecified laterality, with sciatica presence unspecified, sciatica laterality unspecified; Chronic left-sided low back pain with bilateral sciatica; Chronic right-sided low back pain with bilateral sciatica; Left low back pain, unspecified chronicity, with sciatica presence unspecified; Left LBP; Low back pain without sciatica, unspecified back pain laterality, unspecified chronicity; Acute left-sided low back pain, with sciatica presence unspecified; Low back pain, unspecified back pain laterality, with sciatica presence unspecified; Chronic low back pain; Acute midline low back pain, unspecified whether sciatica present; Low back pain of multiple sites of spine with sciatica; Low back pain radiating to right leg; Pregnancy related low back pain, antepartum, first trimester; Low back pain radiating to left lower extremity; Midline low back pain, unspecified chronicity, with sciatica presence unspecified; Acute right-sided low back pain, with sciatica presence unspecified; Pregnancy with low back pain, antepartum, third trimester; LBP radiating to both legs; Chronic low back pain without sciatica, unspecified back pain laterality; Acute low back pain without sciatica, unspecified back pain laterality; Low back pain with bilateral sciatica; Low back pain radiating to right lower extremity; Acute mechanical low back pain with duration of less than six weeks; Acute bilateral low back pain; Chronic lower back pain; Chronic right-sided low back pain without sciatica; Acute midline low back pain with right-sided sciatica; Low back pain associated with a spinal disorder other than radiculopathy or spinal stenosis; Acute low back pain with disc symptoms, duration less than 6 weeks; Low back pain associated with a spinal disorder other than radiculopathy or spinal stenosis, unspecified laterality, with sciatica presence unspecified, sciatica laterality unspecified; Right low back pain, unspecified chronicity, unspecified whether sciatica present; Lower thoracic back pain; Left-sided low back pain with right-sided sciatica, unspecified chronicity; Chronic bilateral low back pain, unspecified whether sciatica present; Low back pain with radiation, right; Right low back pain, with sciatica presence unspecified; Other intervertebral disc degeneration, lumbar region without mention of lumbar back pain or lower extremity pain; Acute low back pain with right-sided sciatica; Lumbar back pain with radiculopathy affecting left lower extremity; Low back pain with sciatica, sciatica laterality unspecified, unspecified back pain laterality, unspecified chronicity; Degeneration of intervertebral disc of lumbar region without discogenic back pain or lower extremity pain; Degeneration of intervertebral disc of lumbosacral region with discogenic back pain; Acute left-sided low back pain with right-sided sciatica; Acute bilateral low back pain with sciatica; Low back pain with neuralgia of left sciatic nerve; Acute bilateral low back pain with right-sided sciatica; Other intervertebral disc degeneration, lumbosacral region with discogenic back pain only; Acute right-sided low back pain; Bilateral low back pain; Back pain of lumbar region with sciatica; Acute low back pain due to spinal disorder; Low back pain with sciatica, unspecified laterality, sciatica laterality unspecified; Chronic left-sided low back pain with right-sided sciatica; Acute midline low back pain; Low back pain during pregnancy in third trimester; Discogenic low back pain; Right-sided low back pain with bilateral sciatica; Episodic low back pain; Pregnancy with low back pain in second trimester, antepartum; Chronic right-sided low back pain with left-sided sciatica; Midline low back pain, unspecified chronicity, unspecified whether sciatica present; Low back pain, postural; Acute left-sided low back pain; Lt low back pain; Right-sided low back pain with sciatica, sciatica laterality unspecified, unspecified chronicity; Pregnancy with low back pain in third trimester, antepartum; Midline low back pain with right-sided sciatica, unspecified chronicity; Low back pain with radiation; Left low back pain; Low back pain with right-sided sciatica, unspecified back pain laterality; Low back pain without sciatica, unspecified; Chronic right-sided low back pain, with sciatica presence unspecified; Chronic low back pain with left-sided sciatica, unspecified back pain laterality; Midline low back pain with bilateral sciatica, unspecified chronicity; Right-sided low back pain with bilateral sciatica, unspecified chronicity; Acute low back pain with sciatica, sciatica laterality unspecified, unspecified back pain laterality; Acute left-sided low back pain with sciatica, sciatica laterality unspecified; Lumbar back pain with radiculopathy affecting right lower extremity; Pregnancy related low back pain, antepartum, unspecified trimester; Chronic low back pain with left-sided sciatica; Chronic LBP; Left-sided low back pain with left-sided sciatica, unspecified chronicity; Low back pain, posterior compartment; Acute radicular low back pain; Back pain of lumbosacral region with sciatica; Acute left-sided low back pain without sciatica; Low back pain with sciatica; Posterior compartment low back pain; Lower back pain; Non-specific low back pain; Midline low back pain with right-sided sciatica; Right-sided low back pain with sciatica; Right-sided low back pain with left-sided sciatica; Other intervertebral disc degeneration, lumbosacral region without mention of lumbar back pain or lower extremity pain; Other intervertebral disc degeneration of lumbosacral region without lumbar back pain or lower extremity pain; Radicular low back pain; Acute midline low back pain with sciatica, sciatica laterality unspecified; Acute low back pain with sciatica; Right-sided low back pain with right-sided sciatica, unspecified chronicity; Midline low back pain with left-sided sciatica; Acute low back pain with left-sided sciatica; Other intervertebral disc degeneration, lumbar region with discogenic back pain only; Acute low back pain, unspecified back pain laterality, unspecified whether sciatica present; Low back pain, episodic; Pregnancy related low back pain, antepartum, third trimester; Low back pain with left-sided sciatica, unspecified back pain laterality, unspecified chronicity; Low back pain during pregnancy, unspecified trimester; Midline low back pain with left-sided sciatica, unspecified chronicity; Acute left-sided low back pain, unspecified whether sciatica present; Midline low back pain, with sciatica presence unspecified; Chronic bilateral low back pain; Chronic left-sided low back pain, with sciatica presence unspecified; Midline low back pain without sciatica; Lower back pains; Acute lumbar back pain; Low back pain, unspecified; Midline low back pain with sciatica; Postural low back pain; Acute midline low back pain with left-sided sciatica; Left-sided low back pain without sciatica, unspecified chronicity; Bilateral low back pain, unspecified chronicity, unspecified whether sciatica present; Low back pain, recurrent; Acute bilateral low back pain with left-sided sciatica; Low back pain, unspecified back pain laterality, unspecified chronicity, with sciatica presence unspecified; Low back pain at multiple sites; Vertebrogenic low back pain; Acute low back pain; Acute right-sided low back pain with left-sided sciatica; Acute right-sided low back pain without sciatica; Acute midline low back pain without sciatica; Acute low back pain with symptoms of intervertebral disc disorder, duration less than 6 weeks; Chronic low back pain with bilateral sciatica, unspecified back pain laterality; Low back pain, mechanical; Lumbar back pain; Pregnancy with low back pain, antepartum, unspecified trimester; Low back pain with sciatica, right; Low back pain with sciatica, sciatica laterality unspecified, unspecified back pain laterality; Midline low back pain without sciatica, unspecified chronicity; Pregnancy with low back pain, antepartum, first trimester; Low back pains; LBP (low back pain); Back pain, lower; Acute midline low back pain, with sciatica presence unspecified; Right low back pain; Back pains, low; Bilateral low back pain with sciatica, sciatica laterality unspecified, unspecified chronicity; Midline low back pain with sciatica, sciatica laterality unspecified, unspecified chronicity; Chronic left-sided low back pain; Low back pain on left side with sciatica; Left low back pain, unspecified chronicity, unspecified whether sciatica present; Myofascial low back pain; Other intervertebral disc degeneration, lumbar region with discogenic back pain and lower extremity pain; Acute low back pain with bilateral sciatica; Acute low back pain with right-sided sciatica, unspecified back pain laterality; Right-sided low back pain with left-sided sciatica, unspecified chronicity; Left-sided low back pain with sciatica, sciatica laterality unspecified, unspecified chronicity; Low back pain with left-sided sciatica, unspecified; Bilateral low back pain with bilateral sciatica, unspecified chronicity; Low back pain with bilateral sciatica, unspecified back pain laterality, unspecified chronicity; Low back pain during pregnancy in first trimester; Chronic midline low back pain with left-sided sciatica; Left-sided low back pain with bilateral sciatica; Bilateral low back pain with right-sided sciatica, unspecified chronicity; Midline low back pain with bilateral sciatica; Acute bilateral low back pain with bilateral sciatica; Chronic midline low back pain with sciatica, sciatica laterality unspecified; Chronic left-sided low back pain with sciatica; Low back pain due to bilateral sciatica; Low back pain with right-sided sciatica, unspecified; Low back pain with radiation, unspecified laterality; Low back pain radiating to both legs; Acute low back pain due to trauma; Chronic bilateral low back pain with sciatica; Severe LBP; Right-sided low back pain without sciatica; Low back pain with radiation, left; Chronic low back pain, unspecified back pain laterality, unspecified whether sciatica present; Low back pain during pregnancy, antepartum; Acute right-sided low back pain with sciatica; Bilateral low back pain without sciatica; Low back pain, non-specific; Left low back pain, with sciatica presence unspecified; Low back pain potentially associated with spinal stenosis, unspecified laterality, with sciatica presence unspecified, sciatica laterality unspecified; Low back pain, unspecified, with sciatica presence unspecified; Pregnancy related low back pain, antepartum, second trimester; Bilateral low back pain, unspecified chronicity, with sciatica presence unspecified; Acute bilateral low back pain without sciatica; Bilateral low back pain with left-sided sciatica, unspecified chronicity; Acute low back pain with radicular symptoms, duration less than 6 weeks; Low back pain without sciatica; Acute left-sided low back pain with bilateral sciatica; Acute right-sided low back pain with bilateral sciatica; Chronic left-sided low back pain with left-sided sciatica; Chronic bilateral low back pain with bilateral sciatica; Chronic midline low back pain with bilateral sciatica; Acute low back pain with bilateral sciatica, unspecified back pain laterality; Chronic midline low back pain with right-sided sciatica; Other intervertebral disc degeneration, lumbosacral region with discogenic back pain and lower extremity pain; Back pain of thoracolumbar region; Low back pain due to displacement of intervertebral disc; Low back pain with right-sided sciatica; Other intervertebral disc degeneration of lumbar region without lumbar back pain or lower extremity pain; Chronic radicular low back pain; Degeneration of intervertebral disc of lumbosacral region with discogenic back pain and lower extremity pain; Autosomal recessive retinitis pigmentosa associated with mutation in RLBP1 gene; Intractable low back pain; Chronic bilateral low back pain, with sciatica presence unspecified; Complains of low back pain; Traumatic low back pain; Bilateral low back pain with sciatica, sciatica laterality unspecified; Acute low back pain with possible spinal stenosis of less than six weeks' duration; Biallelic mutation of RLBP1 gene; Chronic midline low back pain, unspecified whether sciatica present; Recurrent low back pain; Back pains, lower; Low back pain during pregnancy; Pregnancy related low back pain in third trimester, antepartum; Pregnancy with low back pain, antepartum; Pregnancy related low back pain, antepartum; LBP radiating to left leg; Low back pain of over 3 months duration; Right LBP; Multifactorial low back pain; Left-sided low back pain with sciatica, sciatica laterality unspecified; RLBP1-related retinitis pigmentosa, autosomal recessive; H/O low back pain; Back pain, low; Low back pain radiating to left leg; Right-sided low back pain without sciatica, unspecified chronicity; Low back pain with sciatica, sciatica laterality unspecified, unspecified; Low back pain during pregnancy in second trimester; Low back pain without sciatica, unspecified back pain laterality; Right-sided low back pain with sciatica, sciatica laterality unspecified; Low back pain; Low back pain of thoracolumbar region with sciatica; Low back pain potentially associated with radiculopathy, unspecified laterality, with sciatica presence unspecified, sciatica laterality unspecified; Low back pain with left-sided sciatica; Low back pain potentially associated with radiculopathy; Pregnancy related low back pain in first trimester, antepartum; Chronic midline low back pain; Acute exacerbation of chronic low back pain; Lumbar back pain with radiculopathy affecting lower extremity; Pregnancy with low back pain, antepartum, second trimester; History of low back pain; Acute left-sided low back pain with sciatica; Acute bilateral low back pain with sciatica, sciatica laterality unspecified; Acute low back pain with left-sided sciatica, unspecified back pain laterality; Acute right-sided low back pain with right-sided sciatica; Acute midline low back pain with sciatica; Family history of low back pain; Right-sided low back pain with right-sided sciatica; Chronic bilateral low back pain with right-sided sciatica; Left-sided low back pain with bilateral sciatica, unspecified chronicity; Chronic primary low back pain; Low back pain radiating to lower extremity; Low back pain with right-sided sciatica, unspecified back pain laterality, unspecified chronicity; Low back pain during pregnancy, first trimester; Pregnancy with low back pain in first trimester, antepartum; Acute right-sided low back pain with sciatica, sciatica laterality unspecified; Pregnancy related low back pain in second trimester, antepartum; Bilateral low back pain with right-sided sciatica; Chronic bilateral low back pain with sciatica, sciatica laterality unspecified; Chronic right-sided low back pain with sciatica, sciatica laterality unspecified; Chronic low back pain with sciatica; Chronic right-sided low back pain with right-sided sciatica; Acute low back pain, unspecified back pain laterality, with sciatica presence unspecified; Acute mechanical low back pain, duration < 6 weeks; Back pain of lumbosacaral region with sciatica; Low back pain with left-sided sciatica, unspecified back pain laterality; Degeneration of intervertebral disc of lumbar region with discogenic back pain and lower extremity pain; Thoracolumbar back pain; Midline low back pain; Chronic low back pain with bilateral sciatica; Low back pain syndrome; Low back pain during pregnancy, third trimester; Back pain, lumbosacral; Acute left-sided low back pain with left-sided sciatica; Acute on chronic low back pain; Left-sided low back pain with left-sided sciatica; Bilateral low back pain with left-sided sciatica; Left-sided low back pain with sciatica; Low back pain on right side with sciatica; Left-sided low back pain with right-sided sciatica; Acute midline low back pain with bilateral sciatica; Chronic low back pain with sciatica, sciatica laterality unspecified, unspecified back pain laterality; Bilateral low back pain without sciatica, unspecified chronicity; Low back pain with sciatica, left; Mechanical low back pain; Acute bilateral low back pain, unspecified whether sciatica present; Chronic midline low back pain without sciatica; Chronic low back pain without sciatica; Chronic bilateral low back pain without sciatica; Bilateral low back pain with sciatica; Degeneration of intervertebral disc of lumbar region with discogenic back pain

# **Supplemental Code Snippet 3: Patient exclusion criteria**

SQL queries of the de-identified electronic health record.

```
# Age
bday_exclusion_query = f"""
        SELECT
          i.patientdurablekey,
          i.startdatekey,
          p.birthdate,
          DATE_ADD(p.birthdate, INTERVAL 18 YEAR) AS date_18_years
        FROM query2_LBP_df as i
        JOIN patdurabledim as p
        ON
          i.patientdurablekey = p.patientdurablekey
        WHERE
          i.startdatekeyvalue < DATE_ADD(p.birthdate, INTERVAL 18 YEAR)
        ORDER BY
            birthdate DESC
"""

# Procedures
surgery_list = ['DISCECTOMY','LAMINECTOMY','LAMINOTOMY','FORAMINOTOMY','FACETECTOMY']
surgery_abbrev_list = ['ALIF','ASF','PSF','OLIF','TLIF','XLIF']

surgery_query_txt = ' OR '.join(['procedurename LIKE \'%'+x+'%\'' for x in surgery_list])
surgery_abbrev_query_txt = ' OR '.join(['UPPER(procedurename) LIKE \'%'+x+'%\'' for x in surgery_abbrev_list])

surgery_exclusion_query = f'''
/*
Description: Select relevant surgeries (fusion or surgeries for spine or interbody) and
injections (injections, facet and nerve blocks, radiofrequency ablation)
*/

SELECT DISTINCT
  patientdurablekey,
  procedurekey,
  procedurename,
  procedurecode,
  procedurecategory,
  procedurestartdatekey,
  procedureenddatekey,
  CASE
      WHEN UPPER(procedurename) LIKE '%INJECTION%' THEN 'INJECTION'
      WHEN UPPER(procedurename) LIKE '%BLOCK%' THEN 'BLOCK'
      WHEN UPPER(procedurename) LIKE '%ABLATION%' THEN 'RADIOFREQUENCY ABLATION'
      WHEN UPPER(procedurename) LIKE '%ARTHRODESIS%' THEN 'FUSION'
      WHEN UPPER(procedurename) LIKE '%FUSION%' THEN 'FUSION'
      WHEN UPPER(procedurename) LIKE '%ALIF%' THEN 'FUSION'
      WHEN UPPER(procedurename) LIKE '%ASF%' THEN 'FUSION'
      WHEN UPPER(procedurename) LIKE '%PSF%' THEN 'FUSION'
      WHEN UPPER(procedurename) LIKE '%OLIF%' THEN 'FUSION'
      WHEN UPPER(procedurename) LIKE '%TLIF%' THEN 'FUSION'
      WHEN UPPER(procedurename) LIKE '%XLIF%' THEN 'FUSION'
      WHEN UPPER(procedurename) LIKE '%SURGERY%' THEN 'SURGERY'
      WHEN UPPER(procedurename) LIKE '%OSTEOTOMY%' THEN 'OSTEOTOMY'
      ELSE 'SURGERY'
      END AS proceduretype
FROM
  procedureeventfact
  WHERE
    patientdurablekey IN {tuple(patientdurablekey_workinglist)}

    AND
    (
        --FOR surgery (specific names)
        {surgery_query_txt}
        OR
        --FOR fusions (full name)
        (
            (
            UPPER(procedurename) LIKE '%FUSION%'
            OR UPPER(procedurename) LIKE '%ARTHRODESIS%'
            )
            AND
            (
            UPPER(procedurename) LIKE '%SPINE%'
            OR UPPER(procedurename) LIKE '%INTERBODY%'
            OR UPPER(procedurename) LIKE '%LUMB%'
            )
        )
        OR
        --FOR fusions (abbreviations)
        (
            (
            {surgery_abbrev_query_txt}
            )
            AND
            (
            UPPER(procedurename) LIKE '%SPINE%'
            OR UPPER(procedurename) LIKE '%INTERBODY%'
            OR UPPER(procedurename) LIKE '%LUMBAR%'
            OR UPPER(procedurename) LIKE '%FUSION%'
            )
        )
        OR
        --FOR surgeries
        (
            (
            UPPER(procedurename) LIKE '%SURGERY%'
            OR UPPER(procedurename) LIKE '%OSTEOTOMY%'
            )
            AND
            (
            UPPER(procedurename) LIKE '%SPINE%'
            OR UPPER(procedurename) LIKE '%DISC %' -- not DISCharge, lumbar/cervical/thoracic disc surgery
            OR UPPER(procedurename) like '%LUMB%'
            )
        )
        OR
        -- FOR injection
        (
            (
            UPPER(procedurename) LIKE '%INJECTION%' -- includes epidural injections
            OR UPPER(procedurename) LIKE '%BLOCK%'
            OR  (UPPER(procedurename) LIKE '%ABLATION%'
                AND UPPER(procedurename) LIKE '%RADIOFREQUENCY%'
                )
            )
            AND UPPER(procedurename) LIKE '%LUMB%'
        )
    )
ORDER BY
    patientdurablekey,
    procedurestartdatekey

'''

# Confounders of LBP
#infection in bone, intervertebral disc, vertebral endplate and vertebral body, disc and adjacent vertebral body, disc and adjacent vertebral body
exclude_diag_list = ['osteomyelitis','discitis','spondylitis','spondylodiscitis','septic facet joint']
exclude_diag_query_txt = ' OR '.join(['LOWER(diagnosisname) LIKE \'%'+x+'%\'' for x in exclude_diag_list])

diagnosis_exclusion_query = f'''
/*
Description: Select confounder of LBP: fracture, stroke, cancer, infection, trauma.
*/

SELECT
  patientdurablekey,
  diagnosiseventkey,
  diagnosiskey,
  diagnosisname,
  encounterkey,
  startdatekey,
  enddatekey,
  CASE
  WHEN LOWER(diagnosisname) LIKE '%fracture%' THEN 'fracture'
  WHEN LOWER(diagnosisname) LIKE '%failed back%' THEN 'failed back'
  WHEN LOWER(diagnosisname) LIKE '%abscess%' THEN 'abscess'
  WHEN LOWER(diagnosisname) LIKE '%lymph%' THEN 'lymph'
  WHEN LOWER(diagnosisname) LIKE '%stroke%' THEN 'stroke'
  WHEN LOWER(diagnosisname) LIKE '%sepsis%' THEN 'sepsis'
  WHEN LOWER(diagnosisname) LIKE '%paresis%' THEN 'paresis'
  WHEN ({exclude_diag_query_txt}) THEN 'infection'
  WHEN (LOWER(diagnosisname) LIKE '%tumor%' OR LOWER(diagnosisname) LIKE '%metastasis%' OR LOWER(diagnosisname) LIKE '%oncology%') THEN 'cancer'
  WHEN LOWER(diagnosisname) LIKE '%infection%' THEN 'infection'
  WHEN LOWER(diagnosisname) LIKE '%trauma%' THEN 'trauma'
  ELSE 'category not specified'
  END AS diagnosistype
FROM
  diagnosiseventfact

  WHERE
  patientdurablekey IN {tuple(patientdurablekey_workinglist)}
  AND
  (
    -- diagnosis exclusions
    {exclude_diag_query_txt}

    OR LOWER(diagnosisname) LIKE '%fracture%'
    OR LOWER(diagnosisname) LIKE '%failed back%'
    OR LOWER(diagnosisname) LIKE '%abscess%'

    OR LOWER(diagnosisname) LIKE '%tumor%'
    OR LOWER(diagnosisname) LIKE '%metastasis%'
    OR LOWER(diagnosisname) LIKE '%oncology%'
    OR LOWER(diagnosisname) LIKE '%lymph%'
    OR LOWER(diagnosisname) LIKE '%stroke%'
    OR LOWER(diagnosisname) LIKE '%sepsis%'
    OR LOWER(diagnosisname) LIKE '%paresis%'
    OR LOWER(diagnosisname) LIKE '%spondyloarthr%'
    OR LOWER(diagnosisname) LIKE '%infection%'
    OR LOWER(diagnosisname) LIKE '%trauma%'
  )

  ORDER BY
    diagnosiskey,
    patientdurablekey
'''
```
